# Post-marketing surveillance to assess the safety and tolerability of a combined diphtheria, tetanus, acellular pertussis and inactivated poliovirus vaccine (DTaP-IPV) in Korean children

**DOI:** 10.1080/21645515.2019.1572406

**Published:** 2019-03-19

**Authors:** Soon Min Lee, Sung Jin Kim, Jing Chen, Rok Song, Joon-Hyung Kim, Raghavendra Devadiga, Yun-Kyung Kim

**Affiliations:** a Department of Pediatrics, Gangnam Severance Hospital, Yonsei University College of Medicine, Gangnam-gu, Seoul, Republic of Korea; b GSK, LS Yong-san Tower, Hangang-daero, Yongsan-gu, Seoul, Republic of Korea; c GSK, Singapore, Singapore; d GSK, CDOC-B, Bangalore, India; e Department of Pediatrics, Korea University College of Medicine, Seoul, Republic of Korea

**Keywords:** (5-10): DTaP-IPV, Post-marketing surveillance, Safety, Korea, Diphtheria, Tetanus, Pertussis, Poliomyelitis

## Abstract

*Infanrix-IPV* (GSK, Belgium) is a diphtheria, tetanus, acellular pertussis, and inactivated poliovirus combination vaccine (DTaP-IPV) licensed in many countries including Korea. In accordance with Korean regulations, we conducted a post-marketing surveillance (PMS) to evaluate the safety of DTaP-IPV administered to Korean children in routine immunization schedules. Children aged <7 years receiving at least one dose of DTaP-IPV either as part of a primary (3-dose) vaccination series or as a subsequent booster were enrolled. Adverse events (AEs), adverse drug reactions (ADRs) and serious AEs (SAEs) were recorded after each dose during the 30-day post-vaccination follow-up period. Among a total of 639 children, 289 subjects (45.2%) experienced AEs, mostly (79.2%) assessed as being unlikely to be related to the vaccination. ADRs were reported in 13.0% of subjects. Fever was the most commonly reported expected AE (11.9% of subjects) and also the most commonly reported expected ADR (8.5% of subjects). No obvious association between AE incidence and vaccine dose sequence was apparent. An unexpected AE was seen in 32.9% of children, and unexpected ADRs were far less common (1.9%). Thirty-four SAEs were recorded in 26 subjects (4.1%), in two of whom a causal association with the vaccine could not be excluded, although both resolved quickly. Data from this PMS indicate that DTaP-IPV has an acceptable safety profile when given to Korean children in accordance with local prescribing recommendations in routine childhood immunization. ClinicalTrials.gov identifier: NCT01568060

## Introduction

Globally, diphtheria, tetanus, pertussis (or whooping cough) and polio cause significant morbidity and mortality particularly amongst children.^1^ In Korea, childhood vaccinations against diphtheria, tetanus, and pertussis (DTaP) and against polio have been included in the National Immunization Program (NIP) for over 30 years.^2^ Recommended immunization schedules include giving DTaP as a three-dose primary vaccination series in infants aged 2, 4 and 6 months, with a fourth dose given at 15–18 months, and a fifth dose at 4–6 years of age.^3^ For polio, primary vaccination at age 2, 4 and 6 months is recommended, with a subsequent booster dose at 4–6 years.^3^ High rates of vaccine uptake are reported; surveillance data for 2012 report that for DTaP, 99.1% of children completed primary vaccination, while 83.6% received a fourth and 56.6% a fifth dose.^4^ In 2013, 99.6% of children completed primary series of DTaP and 96.8% received a fourth DTaP dose.^5^ For polio, reported vaccine uptake in 2012 was 98.3% and 73.0% for primary and booster vaccinations, respectively.^4^

A combined diphtheria, tetanus, acellular pertussis and inactivated poliovirus vaccine (DTaP-IPV, *Infanrix-IPV*, GSK, Belgium) has been available for use in Korea since 2012.^6^ *Infanrix-IPV* is indicated for use in a three-dose primary schedule for immunization of infants aged 2 months and older, against diphtheria, tetanus, pertussis and poliomyelitis. DTaP-IPV is also indicated for use as the fifth dose of the DTaP vaccine series and the fourth dose of the IPV series in children aged 4–6 years.^6^ By reducing overall total number of vaccine administrations and clinical visits, combination vaccines offer a number of advantages when implementing often crowded childhood immunization schedules. Advantages include greater flexibility and convenience, with potentially improved vaccine uptake and reduced costs.^7^ Data from a randomized controlled trial (RCT) conducted in Korean infants indicate that DTaP-IPV has comparable immunogenicity and safety to that seen with separate DTaP and IPV vaccinations when given as a three-dose primary vaccination schedule.^8^ Studies conducted in other populations (including the United States [US], Europe, and Australia) also indicate comparable immunogenicity and safety to separate DTaP and IPV vaccinations when given a booster-dose. In addition, they show DTaP-IPV can be safely co-administered with other childhood vaccinations,^9–15^ and post-licensure surveillance data from the US indicate no safety concerns.^16^

In accordance with regulations from the Ministry of Food and Drug Safety (MFDS) in Korea,^17^ additional safety information on the use of licensed vaccines in the Korean population is required following vaccine registration.^18,19^ We conducted a post-marketing surveillance (PMS) to evaluate the safety of the DTaP-IPV vaccine when administered according to the local prescribing information (PI) in children in Korea (NCT01568060). The main objectives were to document all adverse events (AEs) occurring during the 30-day (day 0 to day 29) follow-up period after each vaccine dose, including those with unestablished causal relationships to the study vaccine (i.e., unexpected AEs), and all serious adverse events (SAEs) occurring throughout the study (i.e., from receiving the first dose of the vaccine until 30 days after receiving the last vaccine dose). Those adverse events considered related to the study vaccine, or adverse drug reactions (ADRs), and serious ADRs occurring during the surveillance period were also documented.

## Results

### Study population

In total, 644 subjects were enrolled by 17 doctors in 16 hospitals. Of these, five subjects were excluded from the study’s formal safety analysis: two received DTaP-IPV outside the recommended age (Dose 1 given before 2 months of age), two were of non-Korean ethnicity, and one subject received vaccination in another country (United Kingdom). As such, 639 children were included in the vaccinated cohort for safety analyses of which 342 (53.5%) were male. Demographic data for children in this cohort are shown in Table 1.

**Table 1.** Study population and vaccine doses received.

Parameter	Total N = 639
Male	342 (53.5%)
Body weight, kg	
Median (range)	6.3 (1.5–43.8)
Age when subjects received primary vaccine dose within the PMS	
8–16 weeks	367 (57.4%)
>16–24 weeks	110 (17.2%)
>24 weeks – <48 months	99 (15.5%)
Age when subjects received the booster dose within the PMS	
48–60 months	40 (6.3%)
>60 months	23 (3.6%)
Number of vaccine doses received	
1	302 (47.3%)
2	121 (18.9%)
3	216 (33.8%)
Receiving primary vaccination dose	576 (90.1%)
Dose 1	367 (57.4%)
Dose 2	373 (58.4%)
Dose 3	389 (60.9%)
Receiving booster vaccination dose	63 (9.9%)

All data are presented as number of subjects (%) except where otherwise stated

N, total vaccinated cohort; PMS, post-marketing surveillance

**Figure 1.**
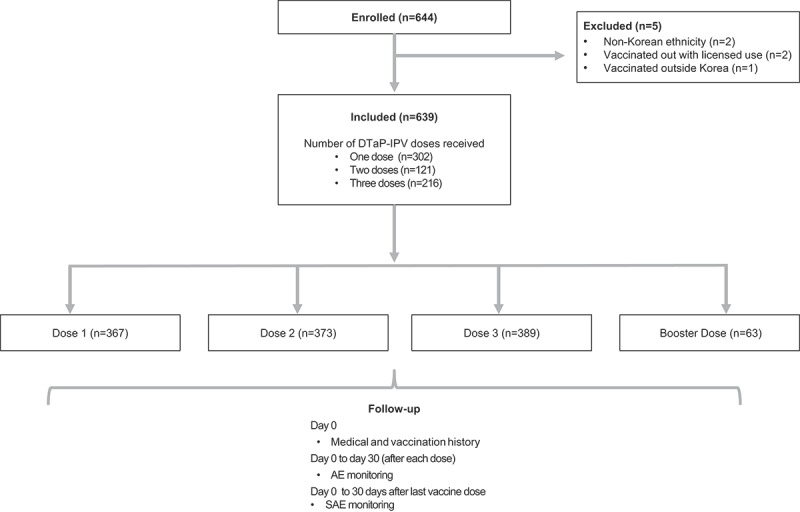
Study flow. AE, adverse event; SAE, serious adverse event

Of the 639 children receiving at least one DTaP-IPV dose within the PMS period, some subjects received more than one vaccine dose; 302 (47.3%) received one dose; 121 (18.9%) received two doses, and 216 subjects (33.8%) received three doses (with a total of 1,192 separate vaccine doses administered within this PMS). When accounting for overlap due to multiple dosing, 576 children (90.1%) received at least one DTaP-IPV dose as part of their primary vaccination series and 63 (9.9%) received a booster dose. From a dosing schedule perspective, the number of subjects receiving the DTaP-IPV primary vaccination sequence was as follows; Dose 1, n = 367 (57.4% of the overall cohort); Dose 2, n = 373 (58.4%); and Dose 3, n = 389 (60.9%). Of these, 367 (57.4%) received their first dose (as Dose 1) aged between 8–16 weeks of age, 110 (17.2%) received their first dose (as Dose 2) between 16–24 weeks of age, and 99 (15.5%) received their first dose (as Dose 3) aged between 24 weeks to 48 months. Of those receiving a booster dose, 40 (6.3%) received it when aged between 48–60 months and 23 (3.6%) were >60 months of age (Figure 1).

A history of vaccination within 30 days prior to DTaP-IPV administration was reported for 242 subjects (37.9%), principally for protection against hepatitis or rotavirus infection. When considering any concomitant vaccination (i.e., from 30 days before the administration of DTaP-IPV until 30 days after the final administration of DTaP-IPV), this was reported in 567 (88.7%) subjects, principally *Haemophilus influenzae* type b, pneumococcal, rotavirus and hepatitis vaccines (Supplementary Table 1).

Two hundred and fifty-six subjects (40.1%) had a history of previous medical illness, including 114 children (17.8%) with conditions arising in the perinatal period and 67 (10.5%) with respiratory conditions. Illness at the time of vaccination was reported in 148 subjects (23.2%), including respiratory conditions in 44 subjects (6.9%), congenital malformations and chromosomal abnormalities in 26 subjects (4.1%), and skin or subcutaneous tissue disorders in 25 subjects (3.9%). Concomitant medication was reported by 317 (49.6%) subjects during the 30-day period following vaccination; cough or cold preparations by 168 (26.3%), allergy medications by 156 (24.4%), analgesics or nonsteroidal anti-inflammatory drugs (NSAIDs) by 135 (21.1%), and anti-infective agents (antibiotics, antifungals or antivirals) by 92 subjects (14.4%) (Supplementary Table 1).

### Safety

All AEs, ADRs, SAEs and serious ADRs were reported after each dose. Overall during the 30-day post-vaccination follow-up period, AEs were reported in 289 subjects (45.2%), who experienced a total of 587 AEs (Table 2). For children receiving DTaP-IPV in the primary vaccination series, the relative number of subjects in whom an AE was reported after each dose was similar: dose 1 (n = 97, 26.4%), dose 2 (n = 113, 30.3%), and dose 3 (n = 113, 29.0%). An AE was reported in 31 children (49.2%) receiving a booster dose (Supplementary Table 2). Most AEs were non-serious (553/587, 94.2% of all AEs), occurring in 263 subjects. Of these, 487/553 (88.1%) were considered by the investigator to be of mild intensity with 59/553 (10.7%) moderate and 7/553 (1.3%) severe. Of these non-serious AEs, six required attendance at the Emergency department, and 289 were treated by medical staff; no other AEs required formal medical care.

**Table 2.** Adverse events (AEs) and adverse drug reactions (ADRs) over the PMS period.

N = 639 (total number of vaccinations = 1,192)					
Adverse Event (AE)			Adverse drug reaction (ADR)		
AE	Subjects n (%, 95% CI)	AE n (%)	ADR	Subjects n (%, 95% CI)	ADR n (%)
At least one AE	289 (45.2; 41.3–49.2)	587	At least one ADR	83 (13.0; 10.5–15.9)	122
Fever	76 (11.9; 9.5–14.7)	87 (14.8%)	Fever	54 (8.5; 6.4–10.9)	59 (48.4%)
Common cold	49 (7.7; 5.7–10.0)	55 (9.4%)	Irritability	15 (2.3; 1.3–3.8)	16 (13.1%)
URTI	46 (7.2; 5.3–9.5)	57 (9.7%)	Injection-site swelling	8 (1.3; 0.5–2.5)	8 (6.6%)
Coughing	24 (3.8; 2.4–5.5)	24 (4.1%)	Injection-site erythema	7 (1.1; 0.4–2.2)	7 (5.7%)
Irritability	23 (3.6; 2.3–5.4)	25 (4.3%)	Injection-site pain	2 (0.3; 0.0–1.1)	2 (1.6%)
Gastroenteritis	23 (3.6; 2.3–5.4)	26 (4.4%)	Fatigue	2 (0.3; 0.1–1.1)	2 (1.6%)
Rhinorrhoea	23 (3.6; 2.3–5.4)	24 (4.1%)	Vomiting	2 (0.3; 0.1–1.1)	2 (1.6%)
Bronchitis	23 (3.6; 2.3–5.4)	23 (3.9%)	Rash	2 (0.3; 0.1–1.1)	2 (1.6%)
Bronchiolitis	22 (3.4; 2.3–5.4)	23 (3.9%)	Erythema	2 (0.3; 0.1–1.1)	2 (1.6%)
Diarrhea	17 (2.7; 1.6–4.2)	19 (3.2%)	Injection-site induration	2 (0.3; 0.0–1.1)	2 (1.6%)
			Injection-site reaction	1 (0.2; 0.0–0.9)	1 (0.8%)
			Kawasaki disease	1 (0.2; 0.0–0.9)	2 (1.6%)

N = total number of infants receiving study vaccine overall (any dose and by specific dose); subject n (%): number (percentage) of subjects with an AE or ADR; AE n (%): number of AEs and (%); ADR n (%): number of ADRs and (%); AE, adverse event; URTI, upper respiratory tract infection.

All % are based on the number of subjects or AEs or ADRs for the respective period.

Subjects may experience more than one AE category.

95% CI: 95% confidence interval; ADR, adverse drug reaction; AE, adverse event; PMS, post-marketing surveillance; URTI, upper respiratory tract infection.

Of the total 587 AEs, 465/587 (79.2%) were considered by the investigators as “unlikely” to be related to the study vaccine, and 122/587 AEs (20.8%) occurring in 83 subjects (13.0%) were considered an ADR (i.e., any AE whose causality due to the study vaccine could not be ruled out). Ten AEs (1.7%) were considered by the investigators as “Certainly” related to the vaccine, with 68 AEs (11.6%) considered “Possibly” related to the vaccine, 36 AEs (6.1%) considered “Probable/Likely” related to the vaccine, 4 AEs (0.7%) considered “Conditional/Unclassified” and 4 AEs (0.7%) considered “Unassessable/Unclassifiable”.

The most frequently reported AEs and ADRs are shown in Table 2 (and Supplementary Table 2). Fever was the most commonly reported AE, with 87/587 cases (14.8% of all AEs) occurring in 76 subjects (11.9% of the study population). Fever was also the most commonly reported ADR, with 59/122 cases (48.4%) occurring in 54 subjects (8.5% of the study population). Injection-site swelling, erythema or pain or other local reaction was relatively uncommon, with a total of 20 injection-site ADRs being reported (16.4% of all ADRs) occurring in 16/639 subjects (2.5% of the study population). Of these; 11 injection-site ADRs occurred in 10/576 subjects (1.7%) who received the vaccine as part of the primary vaccination schedule and nine reactions occurred in 6/63 subjects (9.5%) who received a booster dose.

Of the total AEs reported, 350/587 (59.6%) were considered as unexpected AEs reported in 210/639 subjects (32.9% of the study population). Respiratory or gastrointestinal disorders were most commonly reported; common cold in 49/639 subjects (7.7%), upper respiratory tract infection (URTI) in 46 (7.2%) and gastroenteritis in 23 subjects (3.6%). A total of 17 unexpected AEs occurring in 12 subjects (1.9%) were considered unexpected ADRs (13.9% of all ADRs). Of these, only fatigue was reported in more than one subject (Table 3 and Supplementary Table 3).

**Table 3.** Unexpected adverse events and adverse drug reactions over the PMS period.

N = 639 (total number of vaccinations = 1,192)					
Adverse Event (AE)			Adverse drug reaction (ADR)		
AE	Subjects n (%, 95% CI)	AE n (%)	ADR	Subjects n (%, 95% CI)	ADR n (%)
At least one unexpected AE	210 (32.9; 29.2–36.7)	350	At least one unexpected ADR	12 (1.9; 1.0–3.3)	17
Common cold	49 (7.7; 5.7–10.0)	55 (15.7%)	Fatigue	2 (0.3; 0.0–1.1)	2 (11.8%)
URTI	46 (7.2; 5.3–9.5)	57 (16.3%)	Kawasaki disease	1 (0.2; 0.0–0.9)	2 (11.8%)
Gastroenteritis	23 (3.6; 2.3–5.4)	26 (7.4%)	Dizziness	1 (0.2; 0.0–0.9)	1 (5.9%)
Rhinorrea	23 (3.6; 2.3–5.4)	24 (6.9%)	URTI	1 (0.2; 0.0–0.9)	1 (5.9%)
Bronchiolitis	22 (3.4; 2.2–5.2)	23 (6.6%)	Bronchiolitis	1 (0.2; 0.0–0.9)	1 (5.9%)
Otitis media	15 (2.4; 1.3–3.8)	16 (4.6%)	Laryngitis	1 (0.2; 0.0–0.9)	1 (5.9%)
Pneumonia	10 (1.6; 0.8–2.9)	10 (2.9%)	Tonsillitis	1 (0.2; 0.0–0.9)	1 (5.9%)
			Pharyngitis	1 (0.2; 0.0–0.9)	1 (5.9%)
			Constipation	1 (0.2; 0.0–0.9)	1 (5.9%)
			Flank Pain	1 (0.2; 0.0–0.9)	1 (5.9%)
			Conjunctivitis	1 (0.2; 0.0–0.9)	1 (5.9%)
			Insomnia	1 (0.2; 0.0–0.9)	1 (5.9%)
			Sleep disorder	1 (0.2; 0.0–0.9)	1 (5.9%)
			Feeding disorder in child	1 (0.2; 0.0–0.9)	1 (5.9%)
			Viral infection	1 (0.2; 0.0–0.9)	1 (5.9%)

N = total number of infants receiving study vaccine overall (any dose and by specific dose); subject n (%): number (percentage) of subjects with an AE or ADR; AE n (%): number of AEs and (%); ADR n (%): number of ADRs and (%).

All % are based on the number of subjects or AEs or ADRs for the respective period.

Subjects may experience more than one AE category.

95% CI: 95% confidence interval; ADR, adverse drug reaction; AE, adverse event; PMS, post-marketing surveillance; URTI, upper respiratory tract infection.

Of the 587 AEs reported, 34 (5.8%) were categorized as SAEs, reported in 26 subjects (4.1% of the overall study population). The most commonly reported SAEs were eight cases of bronchiolitis in eight subjects (1.3%), five cases of gastroenteritis in five subjects (0.8%), four cases of pneumonia in four subjects (0.6%), and colitis or urinary tract disorders, each reported in three subjects (0.5%) (Table 4 and Supplementary Table 4). There were no deaths during this surveillance period.

**Table 4.** Serious adverse events and serious adverse drug reactions over the PMS period.

N = 639 (total number of vaccinations = 1,192)					
Serious Adverse Event (SAE)			Serious Adverse drug reaction (ADR)		
SAE	Subjects n (%, 95% CI)	SAE n (%)	Serious ADR	Subjects n (%, 95% CI)	Serious ADR n (%)
At least one SAE	26 (4.1; 2.7–5.9)	34	At least one serious ADR	2 (0.3; 0.0–1.1)	2
Bronchiolitis	8 (1.3; 0.5–2.5)	8 (23.5%)	Fever	1 (0.2; 0.0–0.9)	1 (50.0%)
Gastrointestinal infection	7 (1.1; 0.4–2.2)	9 (26.5%)	Kawasaki disease	1 (0.2; 0.0–0.9)	1 (50.0%)
Gastroenteritis	5 (0.8; 0.3–1.8)	5 (14.7%)			
Pneumonia	4 (0.6; 0.2–1.6)	4 (11.8%)			
Colitis	3 (0.5; 0.1–1.4)	3 (8.8%)			
URTI	1 (0.2; 0.0–0.9)	1 (2.9%)			
Common cold	1 (0.2; 0.0–0.9)	1 (2.9%)			
Bronchitis	1 (0.2; 0.0–0.9)	1 (2.9%)			
Pharyngotonsillitis	1 (0.2; 0.0–0.9)	1 (2.9%)			
Otitis media	1 (0.2; 0.0–0.9)	1 (2.9%)			
Viral infection	1 (0.2; 0.0–0.9)	1 (2.9%)			
Sepsis	1 (0.2; 0.0–0.9)	1 (2.9%)			
Fever	1 (0.2; 0.0–0.9)	1 (2.9%)			
Urinary tract infection	1 (0.2; 0.0–0.9)	1 (2.9%)			
Cystitis	1 (0.2; 0.0–0.9)	1 (2.9%)			
Pyelonephritis	1 (0.2; 0.0–0.9)	1 (2.9%)			
Kawasaki disease	1 (0.2; 0.0–0.9)	1 (2.9%)			
Cervical lymphadenitis	1 (0.2; 0.0–0.9)	1 (2.9%)			

N = total number of infants receiving study vaccine overall (any dose and by specific dose); subject n (%): number (percentage) of subjects with an SAE or serious ADR; SAE n (%): number of SAEs and (%); Serious ADR n (%): number of Serious ADRs and (%).

All % are based on number of subjects or SAEs or Serious ADRs for the respective period.

Subjects may experience more than one SAE category.

95% CI: 95% confidence interval; ADR, adverse drug reaction; PMS, post-marketing surveillance; SAE, serious adverse event; URTI, upper respiratory tract infection.

Most SAEs were considered unrelated to the study vaccine, although in two cases a causal association could not be ruled out, and so these were considered serious ADRs. They included the occurrence of Kawasaki disease in a male infant, 12 days after the first vaccine dose (as Dose 1), which required hospitalization and treatment with immunoglobulin and aspirin. This subject made a complete recovery after 5 days, and this event was considered by the investigator as “unassessable/unclassifiable” in association with the vaccine. In another subject, fever requiring hospitalization occurred following receipt of a third vaccine dose, which resolved within 48 hours; this was considered as “possibly” related to the vaccine. There were no marked differences in frequency of events (i.e., any AEs or ADRs, unexpected AEs or unexpected ADRs, or SAEs or serious ADRs) after any specific vaccine dose.

Of the five subjects excluded from the safety analysis cohort (due to vaccination outside the licensed indication and use), five AEs were reported in three subjects (fever, vomiting, loose stools, bronchitis and tonsillitis). Of these, two were considered SAEs (bronchitis and tonsillitis) reported in the same subject. None of these AEs/SAEs were considered related to the study vaccine.

### Subject characteristics and overall adverse events

We evaluated the effects of subject-specific characteristics on the incidence of AEs, including that of specific populations as required by MFDS in Korea.^17,20^ In the overall cohort, 4/639 subjects had renal impairment, of which three subjects experienced a total of six AEs. One subject with hydronephrosis experienced four AEs (fever, pneumonia, coughing and otitis media) which required hospitalization, none were considered related to the vaccine. Two subjects each experienced a single AE. Of these, one subject developed fever (requiring hospitalization) so it was considered an SAE, and was categorized as “probably” related to the vaccine, and recorded as a serious ADR.

There were no significant differences by gender, current or past medical history or by previous or concomitant vaccination and reporting of number of subjects with AEs (Table 5). However, AEs were significantly more frequent in children taking concomitant medication (74.1%) compared to those who were not (16.8%; p < 0.0001). AEs were also more frequent in children receiving more than one vaccine dose; 38.7% of children receiving only one dose experienced an AE, while AEs were reported in 53.7% of those receiving two doses and in 49.5% of those receiving three doses (p = 0.006). When evaluating relative frequency of AEs as a proportion of all AEs by number of doses received; 200/587 AEs were reported (34.1%) in those receiving one dose, 158/587 (26.9%) in children receiving two doses and 229/587 (39.0%) in those receiving three doses.

**Table 5.** Subject characteristics and incidence of adverse events (AEs).

Characteristic	Adverse Event n	Subjects with Adverse Event n (%, 95% CI)	P-Value
**Renal impairment**			
Yes (N = 4)	6	3 (75.0; 19.4–99.4)	0.333^a^
No (N = 635)	581	286 (45.0; 41.1–49.0)	
**Gender**			
Male (N = 342)	314	162 (47.4; 42.0–52.8)	0.243
Female (N = 297)	273	127 (42.8; 37.1–48.6)	
**Age**			
Primary vaccination			0.080
8–16 weeks (N = 367)	325	152 (41.4; 36.3–46.7)	
>16–24 weeks (N = 110)	124	58 (52.7; 43.0–62.3)	
>24 weeks – <48 months (N = 99)	80	48 (48.5; 38.3–58.8)	
Booster			0.225
48–60 months (N = 40)	33	22 (55.0; 38.5–70.7)	
>60 months (N = 23)	25	9 (39.1; 19.7–61.5)	
**Previous medical condition**			
Yes (N = 256)	237	115 (44.9; 38.7–51.2)	0.899
No (N = 383)	350	174 (45.4; 40.4–50.6)	
**Current medical condition**			
Yes (N = 148)	152	65 (43.9; 35.8–52.3)	0.715
No (N = 491)	435	224 (45.6; 41.2–50.1)	
**Previous vaccination**			
Yes (N = 242)	201	98 (40.5; 34.3–47.0)	0.057
No (N = 396)	386	191 (48.2; 43.2–53.3)	
**Concomitant vaccination**			
Yes (N = 567)	527	254 (44.8; 40.7–49.0)	0.540
No (N = 72)	60	35 (48.6; 36.7–60.7)	
**Concomitant medication**			
Yes (N = 317)	490	235 (74.1; 68.9–78.9)	<0.0001
No (N = 322)	97	54 (16.8; 12.9–21.3)	
**Number of vaccine doses received**			
1 (N = 302)	200	117 (38.7; 33.2–44.5)	0.006
2 (N = 121)	158	65 (53.7; 44.4–62.8)	
3 (N = 216)	229	107 (49.5; 42.7–56.4)	

^a^ P-values calculated using Fisher exact test

Adverse event n (%): number of AEs; Subjects with Adverse event n (%): number (percentage) of subjects with an AE with 95% confidence intervals.

Subjects may experience more than one AE category.

95% CI: 95% confidence interval; AE, adverse event.

## Discussion

Immunization against diphtheria, tetanus, pertussis and polio are key aspects of childhood immunization strategy in Korea.^4^ As such, real-world data from vaccine use in clinical practice regarding safety are essential to inform and improve vaccine use in eligible children.

**Figure 2.**
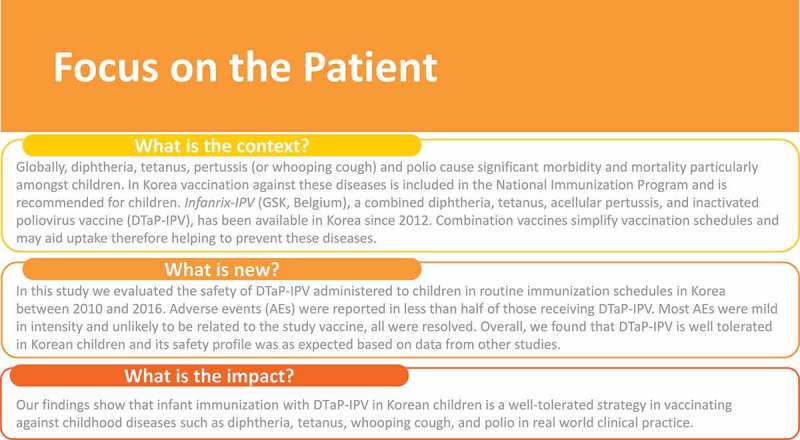
Focus on the patient.

This PMS assessed the safety of the DTaP-IPV vaccine in 639 Korean children when used within routine childhood immunization schedules between 2010 and 2016. We found that the vaccine was well-tolerated with no apparent safety concerns. Figure 2 presents a summary of the context, outcomes, and the impact of this study for health care providers.

AEs were reported in 45.2% of children receiving the study vaccine, although most were mild in intensity. As may be expected, more AEs were reported in those subjects receiving more than one dose of DTaP-IPV. Receiving other vaccines within 30 days of the study vaccine did not influence the frequency of AEs.

The most common expected AE was fever, occurring in 11.9% of subjects, and which accounted for 14.8% of all AEs and 48.4% of all ADRs. This rate is similar to that reported in the pre-licensure RCT investigating immunogenicity and safety in Korean infants (fever ≥37.5°C reported in 11.6% of children^8^) but slightly higher than data from RCTs in non-Korean populations (US, Europe, Australia) that reported rates of fever ranging from 6.1% to 9.6%.^6,14^ Local injection-site reactions were relatively uncommon, with 20 such AEs occurring in 16 subjects (2.5%), most of which were of mild intensity. This is substantially lower than that reported in previous studies in Korean infants (where the proportion of infants with reactions ranged from 17.4% to 32.4%)^8^ and is lower than that reported in previous studies in non-Korean populations.^6,14^

The majority of all AEs reported (59.6%) were considered as unexpected AEs, representing signs/symptoms not described in the Korean PI. Of these, the most frequently reported were respiratory conditions and gastrointestinal infections. While most were considered not vaccine-related, 17 unexpected AEs were considered ADRs accounting for 14% of all ADRs. Most ADRs were isolated cases, although continued monitoring of unexpected ADRs will be important to determine whether these findings are of wider clinical importance.

Over this surveillance period, SAEs were reported in 4.1% of subjects. Most SAEs were unexpected events (bronchiolitis, pneumonia, gastroenteritis or colitis and urinary tract disorders); again, most were assessed as non-vaccine related. Two SAEs were considered serious ADRs i.e., for which the causal relationship to the vaccine could not be ruled out. Both cases required hospitalization, although both resolved quickly. In one case, complete recovery from fever was made within 24 hours without specialized treatment. In the second SAE case, that of Kawasaki disease occurring after receiving the first DTaP-IPV dose, standard treatment (immunoglobulin and aspirin) led to recovery within five days. While the investigator reported this event as “unassessable/unclassifiable” in association with the study vaccine administration (and this infant also received vaccination against rotavirus infection with a pentavalent vaccine), it should be recognized that no clear causal association between vaccination with DTaP-IPV (or any other vaccine) and Kawasaki disease has been found.^21,22^ Furthermore Korea has the second highest incidence of Kawasaki disease in the world (after Japan).^23^ In this case, the infant subsequently received a second dose of DTaP-IPV without any AEs.

Our study has limitations. This was an observational PMS collecting only safety data within routine clinical practice, and the absence of any comparative control group dictates that these data should be interpreted accordingly. Furthermore, our sample size may not be sufficient to detect rare AEs, while comparisons between specific groups may have been influenced by confounders. Nevertheless, our data contribute to existing real-world evidence regarding the acceptable safety of DTaP-IPV in young children.

## Conclusions

In summary, in this PMS conducted over six years in Korean children, we found that DTaP-IPV was well tolerated, with an acceptable safety profile.

## Methods

### Study design and study population

This was a multicenter PMS conducted between June 2010 and June 2016 at 16 hospitals in Korea (NCT01568060). Eligible subjects were children of Korean ethnicity aged between 2 and 83 months who had received at least one dose of DTaP-IPV as part of their routine childhood immunizations (either as part of the primary vaccination schedule or as a subsequent booster dose) in accordance with the Korean PI.^6^ The study was conducted in accordance with MFDS regulatory requirements and the local rules and regulations recruiting a minimum of 600 subjects.^17,20^

### Ethics statement

The study was approved by the independent ethics committee at each participating center. Parents or guardians of subjects provided written informed consent for data collection and analysis before study enrollment.

### DTAP-IPV vaccine

*Infanrix-IPV*, manufactured by GSK, Belgium, comes in a sterile suspension in a single-dose prefilled syringe. Each 0.5 mL vaccine dose contains antigens against the four target pathogens; a minimum of 30 international units (IU) of diphtheria toxoid, 40 IU of tetanus toxoid, three purified antigens of *Bordetella pertussis* (25 μg of pertussis toxoid [PT], 25 μg of filamentous haemagglutinin [FHA] and 8 μg of pertactin [PRN], each adsorbed onto aluminium salt), and three distinct poliovirus antigens; 40 D-antigen units of type 1 poliovirus (Mahoney strain), 8 D-antigen units of type 2 poliovirus (MEF-1 strain) and 32 D-antigen units of type 3 poliovirus (Saukett strain). The vaccine is administered intramuscularly in the thigh (infants ≤2 years) or deltoid (in older children).

### Data collection, safety assessments and AE, ADR and SAE definitions

For all subjects, demographic data, past and current medical history, vaccination history, and data regarding use of concomitant vaccinations/medication were collected at the time of the first vaccine dose within the PMS and updated at any subsequent vaccine dose. Concomitant vaccinations included any other vaccinations administered from 30 days before the administration of the first dose of DTaP-IPV within the PMS until 30 days after the final administration of DTaP-IPV. Concomitant medication was defined as any medication (excluding vitamins) given within a 30-day period prior to or following administration of each vaccine dose (including medications administered to treat an AE or given prophylactically to prevent any vaccine reaction). At all visits the subjects’ axillary, rectal, oral or tympanic temperature was measured prior to vaccination to ensure administration in the absence of fever (oral, axillary or tympanic temperature ≥37.5°C [99.5°F], or rectal temperature ≥38.0°C [100.4°F]).

Subjects were followed up for a period of 30 days after receiving any DTaP-IPV dose. For each subject, the occurrence of any AEs was recorded by the parent/guardian in a diary card and returned to the investigators who recorded all safety information in case report forms. An AE was defined as any untoward medical occurrence, temporally associated with vaccine administration and coded using the World Health Organization Adverse Reactions Terminology (WHO-ART 092 version). The intensity of AEs was recorded as: mild (easily tolerated by the subject, causing minimal discomfort and not interfering with everyday activities), moderate (sufficiently discomforting to interfere with normal everyday activities; fever >38 and ≤39°C) or severe (prevented normal, everyday activities; fever >39°C).

An SAE was defined as any untoward medical occurrence that either resulted in death, was life-threatening, required hospitalization or prolongation of existing hospitalization, or resulted in disability or incapacity. SAEs were recorded throughout the study (i.e., from receiving the first dose of the study vaccine until 30 days after receiving the last vaccine dose). SAEs were identified by study investigators on the basis of diary cards, case report forms and hospital records (with reporting in accordance with the national PMS SAE reporting system). All subjects were followed up until resolution of the AE/SAE or lost to follow-up. AEs (or SAEs) listed in the Korean PI of the study vaccine were categorized as ‘expected’, and those not listed were categorized as ‘unexpected’. Adverse drug reactions (ADRs) were defined as any AE/SAE whose causality to DTaP-IPV could not be ruled out. Causality was categorized as certain, probable/likely, possible, unlikely, conditional/unclassified and unassessable/unclassifiable using criteria based on MFDS guidance (modified from the World Health Organization-The Uppsala Monitoring Centre [WHO-UMC] causality scale).^20,24^

### Data analysis

This was an observational safety study using a pre-defined sample size (≥600) in line with MFDS regulatory requirements, and as such no formal sample size calculation was performed. All data were anonymized and analyses were performed on the vaccinated cohort comprising all subjects eligible for study inclusion who received at least one vaccine dose. The number of subjects experiencing an AE, SAE, or ADR, and the number experiencing expected or unexpected events in each category were analyzed; incidence rates and exact 95% confidence intervals (CI) were then calculated, for the overall cohort and for those receiving specific doses (Doses 1, 2, and 3, or booster dose). The overall incidence of AEs was also stratified by specific factors (gender, medical conditions, concomitant medications, concomitant vaccination, and number of doses received) and comparisons of incidence between groups performed using the Chi^2^ test or Fisher exact test. No formal statistical adjustment for multiple comparisons was applied. Statistical analyses were performed using SAS software (version 9.4; SAS Institute, Inc., NC, USA).

## Trademark section

Infanrix-IPV is a trade mark owned by or licensed to the GSK group of companies.

## References

[1] Centers for Disease Control and Prevention (CDC) Provisional pertussis surveillance report. 2017 [accessed 2018 7 31]. https://www.cdc.gov/pertussis/surv-reporting.html.

[2] Choe YJ, Kim JW, Park YJ, Jung C, Bae GR. Burden of pertussis is underestimated in South Korea: a result from an active sentinel surveillance system. Jpn J Infect Dis. 2014;67:230–32. 24858616 10.7883/yoken.67.230

[3] Kim JH, Choi EH, Park SE, Kim YJ, Jo DS, Kim YK, Eun BW, Lee J, Lee SY, Lee H, et al Recommended immunization schedule for children and adolescents: immunization guideline (8th edition) released by the Korean pediatric society in 2015. Korean J Pediatr. 2016;59(12):461–65. doi:10.3345/kjp.2016.59.12.461. 28194210 PMC5300909

[4] Choe YJ, Yang JJ, Park SK, Choi EH, Lee HJ Comparative estimation of coverage between national immunization program vaccines and non-NIP vaccines in Korea. J Korean Med Sci. 2013;28(9):1283–88. doi:10.3346/jkms.2013.28.9.1283. 24015031 PMC3763100

[5] Korea Centers for Disease Control and Prevention (KCDC) 2013 Korea national immunization survey [in Korean]. [accessed 2018 5 14]. http://cdc.go.kr/CDC/contents/CdcKrContentView.jsp?cid=71894&menuIds=HOME001-MNU1132-MNU2430-MNU2559-MNU2560.

[6] GSK. INFANRIX-IPV Product Information (Korea) [accessed 2018 7 11]. http://drug.mfds.go.kr/html/bxsSearchDrugProduct.jsp?item_Seq=201003939.

[7] Maman K, Zollner Y, Greco D, Duru G, Sendyona S, Remy V The value of childhood combination vaccines: from beliefs to evidence. Hum Vaccin Immunother. 2015;11(9):2132–41. doi:10.1080/21645515.2015.1044180. 26075806 PMC4635899

[8] Kim CH, Cha SH, Shin SM, Kim CS, Choi YY, Hong YJ, Chey MJ, Kim KN, Hur JK, Jo DS, et al Immunogenicity, reactogenicity and safety of a combined DTPa-IPV vaccine compared with separate DTPa and IPV vaccines in healthy Korean infants. Korean J Pediatr Infect Dis. 2010;17(2):156–68. doi:10.14776/kjpid.2010.17.2.156.

[9] Black S, Friedland LR, Schuind A, Howe B GlaxoSmithKline DTaP- IPV vaccine study group. Immunogenicity and safety of a combined DTaP-IPV vaccine compared with separate DTaP and IPV vaccines when administered as pre-school booster doses with a second dose of MMR vaccine to healthy children aged 4-6 years. Vaccine. 2006;24(35–36):6163–71. doi:10.1016/j.vaccine.2006.04.001. 16759769

[10] Black S, Friedland LR, Ensor K, Weston WM, Howe B, Klein NP Diphtheria-tetanus-acellular pertussis and inactivated poliovirus vaccines given separately or combined for booster dosing at 4–6 years of age. Pediatr Infect Dis J. 2008;27(4):341–46. doi:10.1097/INF.0b013e3181616180. 18316985

[11] Klein NP, Weston WM, Kuriyakose S, Kolhe D, Howe B, Friedland LR, Van Der Meeren O An open-label, randomized, multi-center study of the immunogenicity and safety of DTaP-IPV (Kinrix) co-administered with MMR vaccine with or without varicella vaccine in healthy pre-school age children. Vaccine. 2012;30(3):668–74. doi:10.1016/j.vaccine.2011.10.065. 22064267

[12] Nilsson L, Faldella G, Jacquet JM, Storsaeter J, Silfverdal SA, Ekholm L A fourth dose of DTPa-IPV vaccine given to 4–6 year old children in Italy and Sweden following primary vaccination at 3, 5 and 11–12 months of age. Scand J Infect Dis. 2005;37(3):221–29. doi:10.1080/00365540410020884. 15849057

[13] Marshall H, Nolan T, Roberton D, Richmond P, Lambert S, Jacquet JM, Schuerman L A comparison of booster immunisation with a combination DTPa-IPV vaccine or DTPa plus IPV in separate injections when co-administered with MMR, at age 4-6 years. Vaccine. 2006;24(35–36):6120–28. doi:10.1016/j.vaccine.2006.05.017. 16822597

[14] Weston WM, Klein NP Kinrix: a new combination DTaP-IPV vaccine for children aged 4-6 years. Expert Rev Vaccines. 2008;7(9):1309–20. doi:10.1586/14760584.7.9.1309. 18980534

[15] Jacquet JM, Bégué P, Grimprel E, Reinert P, Sandbu S, Silfverdal SA, Faldella G, Nolan T, Lambert S, Richmond P, et al Safety and immunogenicity of a combined DTPa-IPV vaccine administered as a booster from 4 years of age: a review. Vaccine. 2006;24(13):2440–48. doi:10.1016/j.vaccine.2005.12.009. 16406224

[16] Daley MF, Yih WK, Glanz JM, Hambidge SJ, Narwaney KJ, Yin R, Li L, Nelson JC, Nordin JD, Klein NP, et al Safety of diphtheria, tetanus, acellular pertussis and inactivated poliovirus (DTaP-IPV) vaccine. Vaccine. 2014;32(25):3019–24. doi:10.1016/j.vaccine.2014.03.063. 24699471

[17] Ministry of Food and Drug Safety (MFDS) Regulation on safety of medicinal products, etc. [Ordinance of the Prime Minister No.1089, Aug.21, 2014, Partially revised]. 2014 [accessed 2018 7 11]. http://www.mfds.go.kr/eng/eng/index.do?nMenuCode=128&page=3&mode=view&boardSeq=69740.

[18] Choi NK, Park BJ Adverse drug reaction surveillance system in Korea [in Korean]. J Prev Med Public Health. 2007;40:278–84. 17693730 10.3961/jpmph.2007.40.4.278

[19] Song H, Yim DS Problems within the post-marketing surveillance system in Korea: time for a change. Transl Clin Pharmacol. 2016;24(2):63–65. doi:10.12793/tcp.2016.24.2.63.

[20] Ministry of Food and Drug Safety (MFDS) Standard for re-examination of new drugs, etc. [Notification No.2015–79 (Amended by 2015.10.30)]. 2015 [accessed 2018 7 13]. http://www.mfds.go.kr/eng/eng/index.do?nMenuCode=128&page=1&mode=view&boardSeq=71446.

[21] Esposito S, Bianchini S, Dellepiane RM, Principi N Vaccines and Kawasaki disease. Expert Rev Vaccines. 2016;15(3):417–24. doi:10.1586/14760584.2016.1128329. 26634312

[22] Phuong LK, Bonetto C, Buttery J, Pernus YB, Chandler R, Felicetti P, Goldenthal KL, Kucuku M, Monaco G, Pahud B, et al Kawasaki disease and immunisation: A systematic review. Vaccine. 2017;35(14):1770–79. doi:10.1016/j.vaccine.2016.09.033. 28259442

[23] Uehara R, Belay ED Epidemiology of Kawasaki disease in Asia, Europe, and the United States. J Epidemiol. 2012;22(2):79–85. 22307434 10.2188/jea.JE20110131 PMC3798585

[24] World Health Organization (WHO) The use of the WHO-UMC system for standardised case causality assessment. 2005 [accessed 2018 7 13]. http://www.who.int/medicines/areas/quality_safety/safety_efficacy/WHOcausality_assessment.pdf.

