# Supplementary material for: Post-marketing surveillance to assess the safety and tolerability of a combined diphtheria, tetanus, acellular pertussis and inactivated poliovirus vaccine (DTaP-IPV) in Korean children
Source: Hum Vaccin Immunother. 2019 Mar 19;15(5):1145–53. doi: 10.1080/21645515.2019.1572406 (PMC6605835; doi:10.1080/21645515.2019.1572406)
Supplement: Supplemental Material [file khvi-15-05-1572406-s001.docx]

**Supplementary Material**

**Supplementary Table 1 Medical and vaccination history**

| Parameter | n (%) | Parameter | n (%) |
| --- | --- | --- | --- |
| Vaccination history n (%) |  |  |  |
| Vaccinations received within 30 days prior to DTaP-IPV administration as part of the PMS study | **242 (37.9%)** | **Concomitant vaccination**^a^ | 567 (88.7%) |
| Hepatitis vaccine | 130 (20.3%) | *Haemophilus influenzae* type b vaccine | 496 (77.6%) |
| Rotavirus vaccine | 65 (10.2%) | Pneumococcal vaccine | 481 (75.3%) |
| *Haemophilus influenzae* type b vaccine | 33 (5.2%) | Rotavirus vaccine | 418 (65.4%) |
| Tuberculosis vaccine | 33 (5.2%) | Hepatitis vaccine | 301 (47.1%) |
| Measles vaccine | 21 (3.3%) | Influenza vaccine | 104 (16.3%) |
| Specific immunoglobulins | 19 (3.0%) | Specific immunoglobulins | 33 (5.2%) |
| Pneumococcal vaccine | 18 (2.8%) | Measles vaccine | 15 (2.3%) |
| Influenza vaccine | 13 (2.0%) | Encephalitis vaccine | 5 (0.8%) |
| Varicella zoster vaccine | 10 (1.6%) | Tuberculosis vaccine | 5 (0.8%) |
| Medical history^b^ |  |  |  |
| Past medical conditions | **256 (40.1%)** | **Current medical conditions** | 148 (23.2%) |
| Certain conditions originating in the perinatal period | 114 (17.8%) | Diseases of the respiratory system | 44 (6.9%) |
| Diseases of the respiratory system | 67 (10.5%) | Congenital malformations and chromosomal abnormalities | 26 (4.1%) |
| Certain infectious and parasitic disease | 42 (6.6%) | Diseases of the skin and subcutaneous tissue | 25 (3.9%) |
| Diseases of the skin and subcutaneous tissue | 35 (5.5%) | Diseases of the eye and adnexa | 24 (3.8%) |
| Endocrine, nutritional and metabolic diseases | 32 (5.0%) | Certain conditions originating in the perinatal period | 12 (1.9%) |
| Diseases of the digestive system | 26 (4.1%) | Diseases of the digestive system | 11 (1.7%) |
| Congenital malformations and chromosomal abnormalities | 21 (3.3%) | Endocrine, nutritional and metabolic diseases | 10 (1.6%) |
| Concomitant medication, n (%)^b^ | 317 (49.6%) |  |  |
| Cough and Cold Preparations | 168 (26.3%) |  |  |
| Antihistamines and Antiallergics | 156 (24.4%) |  |  |
| Analgesics or NSAIDs | 135 (21.1%) |  |  |
| Anti-infective agents | 92 (14.4%) |  |  |
| ^a^ Concomitant vaccination is receiving any other vaccine within the period from 30 days before the administration of DTaP-IPV until 30 days after the final administration of DTaP-IPV.  ^b^ Concomitant medications include medication administered within 30 days after vaccine dose (and may include medications used to prevent or treat signs/symptoms of AEs.  All the percentages are calculated on the total number of subjects  DTaP-IPV, diphtheria, tetanus, acellular pertussis, and inactivated poliovirus combination vaccine; n, number of subjects; NSAIDs, nonsteroidal anti-inflammatory drugs; PMS, post marketing surveillance | | | |

##

## Supplementary Table 2 Adverse events (AEs) and adverse drug reactions (ADRs) in study population by scheduled dose

|  | **Adverse Event (AE)** | | | **Adverse drug reaction (ADR)** | | |
| --- | --- | --- | --- | --- | --- | --- |
| **Schedule** | **AE** | **Subjects**  **n (%, 95% CI)** | **AE**  **n (%)** | **ADR** | **Subjects**  **n (%, 95% CI)** | **ADR**  **n (%)** |
| **Post dose 1 (N=367)** |  |  |  |  |  |  |
|  | At least one AE | 97 (26.4; 22.0–31.3) | 159 | At least one ADR | 37 (10.1; 7.2–13.6) | 43 |
|  | Fever | 31 (8.5; 5.8–11.8) | 32 (20.1%) | Fever | 22 (6.0; 3.8–8.9) | 22 (51.2%) |
|  | URTI | 20 (5.5; 3.4–8.3) | 21 (13.2%) | Irritability | 8 (2.2; 1.0–4.3) | 8 (18.6%) |
|  | Irritability | 10 (2.7; 1.3–5.0) | 10 (6.3%) | Injection-site erythema | 2 (0.5; 0.1–2.0) | 2 (4.7%) |
|  | Common cold | 9 (2.5; 1.1–4.6) | 10 (6.3%) | Injection-site swelling | 1 (0.3; 0.0–1.5) | 1 (2.3%) |
|  |  |  |  | Injection-site induration | 1 (0.3; 0.0–1.5) | 1 (2.3%) |
|  |  |  |  | Kawasaki disease | 1 (0.3; 0.0–1.5) | 2 (4.7%) |
| **Post dose 2 (N=373)** |  |  |  |  |  |  |
|  | At least one AE | 113 (30.3; 25.7–35.2) | 184 | At least one ADR | 29 (7.8; 5.3–11.0) | 32 |
|  | Fever | 30 (8.0; 5.5–11.3) | 31 (16.8%) | Fever | 21 (5.6; 3.5–8.5) | 21 (65.6%) |
|  | URTI | 21 (5.6; 3.5–8.5) | 22 (12.0%) | Irritability | 6 (1.6; 0.6–3.5) | 6 (18.8%) |
|  | Common cold | 13 (3.5; 1.9–5.9) | 13 (7.1%) | Injection-site swelling | 2 (0.5; 0.1–1.9) | 2 (6.3%) |
|  | Coughing | 10 (2.7; 1.3–4.9) | 10 (5.4%) |  |  |  |
|  | Rhinorrhoea | 10 (2.7; 1.3–4.9) | 10 (5.4%) |  |  |  |
|  | Irritability | 9 (2.4; 1.1–4.5) | 9 (4.9%) |  |  |  |
|  | Bronchitis | 9 (2.4; 1.1–4.5) | 9 (4.9%) |  |  |  |
| **Post dose 3 (N=389)** |  |  |  |  |  |  |
|  | At least one AE | 113 (29.1; 24.6–33.8) | 186 | At least one ADR | 21 (5.4; 3.4–8.1) | 29 |
|  | Common cold | 22 (5.7; 3.6–8.4) | 22 (11.8%) | Fever | 14 (3.6; 2.0–6.0) | 14 (48.3%) |
|  | Fever | 19 (4.9; 3.0–7.5) | 20 (10.8%) | Irritability | 2 (0.5; 0.1–1.8) | 2 (6.9%) |
|  | URTI | 14 (3.6; 2.0–6.0) | 14 (7.5%) | Injection-site erythema | 2 (0.5; 0.1–1.8) | 2 (6.9%) |
|  | Gastroenteritis | 12 (3.1; 1.6–5.3) | 12 (6.5%) | Injection-site swelling | 1 (0.3; 0.0–1.4) | 1 (3.4%) |
|  | Rhinorrhoea | 10 (2.6; 1.2 –4.7) | 10 (5.4%) | Injection-site induration | 1 (0.3; 0.0–1.4) | 1 (3.4%) |
|  | Bronchitis | 8 (2.1; 0.9–4.0) | 8 (4.3%) | Injection-site reaction | 1 (0.3; 0.0–1.4) | 1 (3.4%) |
|  | Coughing | 8 (2.1; 0.9–4.0) | 8 (4.3%) |  |  |  |
|  | Otitis media | 7 (1.8; 0.7–3.7) | 8 (4.3%) |  |  |  |
|  | Irritability | 6 (1.5; 0.6–3.3) | 6 (3.2%) |  |  |  |
|  | Bronchiolitis | 6 (1.5; 0.6–3.3) | 7 (3.8%) |  |  |  |
|  | Diarrhoea | 6 (1.5; 0.6–3.3) | 6 (3.2%) |  |  |  |
| **Post booster (N=63)** |  |  |  |  |  |  |
|  | At least one AE | 31 (49.2; 36.4–62.1) | 58 | At least one ADR | 8 (12.7; 5.7–23.5) | 18 |
|  | Common cold | 10 (15.9; 7.9–27.3) | 10 (17.2%) | Injection-site swelling | 4 (6.4; 1.8–15.5) | 4 (22.2%) |
|  | Injection-site swelling | 4 (6.4; 1.8–15.5) | 4 (6.9%) | Injection-site erythema | 3 (4.8; 1.0–13.3) | 3 (16.7%) |
|  | Fever | 4 (6.4; 1.8–15.5) | 4 (6.9%) | Injection-site pain | 2 (3.2; 0.4–11.0) | 2 (11.1%) |
|  | Tonsillitis | 3 (4.8; 1.0–13.3) | 3 (5.2%) | Fever | 2 (3.2; 0.4–11.0) | 2 (11.1%) |
|  | Pharyngotonsillitis | 3 (4.8; 1.0–13.3) | 3 (5.2%) |  |  |  |
|  | Pruritis | 3 (4.8; 1.0–13.3) | 3 (5.2%) |  |  |  |

N = total number of infants receiving study vaccine overall (any dose and by specific dose); subject n (%): number (percentage) of subjects with an AE or ADR; AE n (%): number of AEs and (%); ADR n (%): number of ADRs and (%).

All % are based on the number of subjects or AEs or ADRs for the respective period.

Subjects may experience more than one AE category.

95% CI: 95% confidence interval; ADR, adverse drug reaction; AE, adverse event; URTI, upper respiratory tract infection.

## Supplementary Table 3 Unexpected adverse events and adverse drug reactions in study population by scheduled dose

|  | **Adverse Event (AE)** | | | **Adverse drug reaction (ADR)** | | |
| --- | --- | --- | --- | --- | --- | --- |
| **Schedule** | **AE** | **Subjects**  **n (%, 95% CI)** | **AE**  **n (%)** | **ADR** | **Subjects**  **n (%, 95% CI)** | **ADR**  **n (%)** |
| **Post dose 1 (N=367)** |  |  |  |  |  |  |
|  | At least one unexpected AE | 62 (16.9; 13.2–21.1) | 90 | At least one unexpected ADR | 4 (1.1;0.3–2.8) | 5 |
|  | URTI | 20 (5.5; 3.4–8.3) | 21 (23.3%) | Kawasaki disease | 1 (0.3; 0.0–1.5) | 2 (40.0%) |
|  | Common cold | 9 (2.5; 1.1–4.6) | 10 (11.1%) | Insomnia | 1 (0.3; 0.0–1.5) | 1 (20.0%) |
|  | Bronchiolitis | 7 (1.9; 0.8–3.9) | 7 (7.8%) | Conjunctivitis | 1 (0.3; 0.0–1.5) | 1 (20.0%) |
|  | Gastroenteritis | 5 (1.4; 0.4–3.2) | 5 (5.6%) | Feeding disorder in child | 1 (0.3; 0.0–1.5) | 1 (20.0%) |
| **Post dose 2 (N=373)** |  |  |  |  |  |  |
|  | At least one unexpected AE | 77 (20.6; 16.7–25.1) | 102 | At least one unexpected ADR | 3 (0.8; 0.2–2.3) | 3 |
|  | URTI | 21 (5.6; 3.5–8.5) | 22 (21.6%) | URTI | 1 (0.3; 0.0–1.5) | 1 (33.3%) |
|  | Common cold | 13 (3.5; 1.9–5.9) | 13 (12.7%) | Fatigue | 1 (0.3; 0.0–1.5) | 1 (33.3%) |
|  | Rhinorrea | 10 (2.7; 1.3–4.9) | 10 (9.8%) | Viral infection | 1 (0.3; 0.0–1.5) | 1 (33.3%) |
| **Post dose 3 (N=389)** |  |  |  |  |  |  |
|  | At least one unexpected AE | 85 (21.9; 17.8–26.3) | 124 | At least one unexpected ADR | 3 (0.8; 0.2–2.2) | 4 |
|  | Common cold | 22 (5.7; 3.6–8.4) | 22 (17.7%) | Pharyngitis | 1 (0.3; 0.0–1.4) | 1 (25.0%) |
|  | URTI | 14 (3.6; 2.0–5.0) | 14 (11.3%) | Laryngitis | 1 (0.3; 0.0–1.4) | 1 (25.0%) |
|  | Gastroenteritis | 12 (3.1; 1.6–5.3) | 12 (9.7%) | Constipation | 1 (0.3; 0.0–1.4) | 1 (25.0%) |
|  | Rhinorrea | 10 (2.6; 1.2–4.7) | 10 (8.1%) | Sleep disorder | 1 (0.3; 0.0–1.4) | 1 (25.0%) |
| **Post booster (N=63)** |  |  |  |  |  |  |
|  | At least one unexpected AE | 25 (39.7; 27.6–52.8) | 34 | At least one unexpected ADR | 3 (4.8; 1.0–13.3) | 5 |
|  | Common cold | 10 (15.9; 7.9–27.3) | 10 (29.4%) | Bronchiolitis | 1 (1.6; 0.0–8.5) | 1 (20.0%) |
|  | Tonsillitis | 3 (4.8; 1.0–13.3) | 3 (8.8%) | Tonsillitis | 1 (1.6; 0.0–8.5) | 1 (20.0%) |
|  | Pharyngotonsillitis | 3 (4.8; 1.0–13.3) | 3 (8.8%) | Fatigue | 1 (1.6; 0.0–8.5) | 1 (20.0%) |
|  | Pharyngitis | 2 (3.2; 0.4–11.0) | 2 (5.9%) | Flank pain | 1 (1.6; 0.0–8.5) | 1 (20.0%) |
|  | Sinusitis | 2 (3.2; 0.4–11.0) | 2 (5.9%) | Dizziness | 1 (1.6; 0.0–8.5) | 1 (20.0%) |
|  | Abdominal pain | 2 (3.2; 0.4–11.0) | 2 (5.9%) |  |  |  |
|  | Bronchiolitis | 1 (1.6; 0.0–8.5) | 1 (2.9%) |  |  |  |
|  | Fatigue | 1 (1.6; 0.0–8.5) | 1 (2.9%) |  |  |  |

N = total number of infants receiving study vaccine overall (any dose and by specific dose); subject n (%): number (percentage) of subjects with an AE or ADR; AE n (%): number of AEs and (%); ADR n (%): number of ADRs and (%).

All % are based on the number of subjects or AEs or ADRs for the respective period.

Subjects may experience more than one AE category.

95% CI: 95% confidence interval; ADR, adverse drug reaction; AE, adverse event; URTI, upper respiratory tract infection.

## Supplementary Table 4 Serious adverse events and serious adverse drug reactions in study population by scheduled dose

|  | **Serious Adverse Event (SAE)** | | | **Serious Adverse drug reaction (ADR)** | | |
| --- | --- | --- | --- | --- | --- | --- |
| **Schedule** | **SAE** | **Subjects**  **n (%, 95% CI)** | **SAE**  **n (%)** | **Serious ADR** | **Subjects**  **n (%, 95% CI)** | **Serious ADR**  **n (%)** |
| **Post dose 1 (N=367)** |  |  |  |  |  |  |
|  | At least one SAE | 10 (2.7; 1.3–5.0) | 16 | At least one serious ADR | 1 (0.3; 0.0–1.5) | 1 |
|  | Bronchiolitis | 4 (1.1; 0.3–2.8) | 4 (25.0%) | Kawasaki disease | 1 (0.3; 0.0–1.5) | 1 (100%) |
|  | Gastroenteritis | 2 (0.5; 0.1–2.0) | 2 (12.5%) |  |  |  |
|  | Colitis | 2 (0.5; 0.1–2.0) | 2 (12.5%) |  |  |  |
|  | Pneumonia | 1 (0.3; 0.0–1.5) | 1 (6.3%) |  |  |  |
|  | URTI | 1 (0.3; 0.0–1.5) | 1 (6.3%) |  |  |  |
|  | Pharyngotonsillitis | 1 (0.3; 0.0–1.5) | 1 (6.3%) |  |  |  |
|  | Sepsis | 1 (0.3; 0.0–1.5) | 1 (6.3%) |  |  |  |
|  | Gastrointestinal infection | 1 (0.3; 0.0–1.5) | 1 (6.3%) |  |  |  |
|  | Urinary tract infection | 1 (0.3; 0.0–1.5) | 1 (6.3%) |  |  |  |
|  | Pyelonephritis | 1 (0.3; 0.0–1.5) | 1 (6.3%) |  |  |  |
|  | Kawasaki disease | 1 (0.3; 0.0–1.5) | 1 (6.3%) |  |  |  |
| **Post dose 2 (N=373)** |  |  |  |  |  |  |
|  | At least one SAE | 10 (2.7; 1.3–4.9) | 12 | At least one serious ADR | 0 (0.0; 0.0-1.0) | 0 |
|  | Bronchiolitis | 3 (0.8; 0.2–2.3) | 3 (25.0%) |  |  |  |
|  | Gastroenteritis | 3 (0.8; 0.2–2.3) | 3 (25.0%) |  |  |  |
|  | Pneumonia | 2 (0.5; 0.1–1.9) | 2 (16.7%) |  |  |  |
|  | Bronchitis | 1 (0.3; 0.0–1.5) | 1 (8.3%) |  |  |  |
|  | Common cold | 1 (0.3; 0.0–1.5) | 1 (8.3%) |  |  |  |
|  | Colitis | 1 (0.3; 0.0–1.5) | 1 (8.3%) |  |  |  |
|  | Viral infection | 1 (0.3; 0.0–1.5) | 1 (8.3%) |  |  |  |
| **Post dose 3 (N=389)** |  |  |  |  |  |  |
|  | At least one SAE | 6 (1.5; 0.6–3.3) | 6 | At least one serious ADR | 1 (0.3; 0.0–1.4) | 1 |
|  | Bronchiolitis | 1 (0.3; 0.0–1.4) | 1 (16.7%) | Fever | 1 (0.3; 0.0–1.4) | 1 (100%) |
|  | Pneumonia | 1 (0.3; 0.0–1.4) | 1 (16.7%) |  |  |  |
|  | Otitis media | 1 (0.3; 0.0–1.4) | 1 (16.7%) |  |  |  |
|  | Fever | 1 (0.3; 0.0–1.4) | 1 (16.7%) |  |  |  |
|  | Cystitis | 1 (0.3; 0.0–1.4) | 1 (16.7%) |  |  |  |
|  | Cervical lymphadenitis | 1 (0.3; 0.0–1.4) | 1 (16.7%) |  |  |  |
| **Post booster (N=63)** |  |  |  |  |  |  |
|  | At least one SAE | - | - | At least one serious ADR | - | - |

N = total number of infants receiving study vaccine overall (any dose and by specific dose); subject n (%): number (percentage) of subjects with an SAE or serious ADR; SAE n (%): number of SAEs and (%); Serious ADR n (%): number of Serious ADRs and (%).

All % are based on number of subjects or SAEs or Serious ADRs for the respective period.

Subjects may experience more than one SAE category.

95% CI: 95% confidence interval; ADR, adverse drug reaction; SAE, serious adverse event; URTI, upper respiratory tract infection
